# Caregiver Perspectives on Electronic Portal Use in Integrated Primary Care: Qualitative Study

**DOI:** 10.2196/84778

**Published:** 2026-08-21

**Authors:** Elizabeth Therese Koval, Tiffany G Munzer, Bryanne M Caldwell, Teryn P Bruni, Sharnita D Harris

**Affiliations:** 1 Indiana School of Medicine Indianapolis, IN United States; 2 University of Michigan School of Medicine Ann Arbor, MI United States; 3 School of Psychology Algoma University Sault Ste. Marie, ON Canada; 4 Nationwide Children's Hospital- Toledo Toledo, OH United States

**Keywords:** electronic patient portal, integrated primary care, developmental behavioral pediatrics, pediatric primary care, health equity, behavioral health

## Abstract

**Background:**

Pediatric patient care portals through electronic health records (EHRs) provide opportunities to enhance patient care. However, challenges exist for making portals usable, and few studies have examined qualitative experiences of caregivers when using integrated primary care (IPC) services for their children.

**Objective:**

This study aimed to qualitatively examine barriers to and facilitators of using electronic patient portals in integrated behavioral health care.

**Methods:**

Data were collected from 7 caregivers of pediatric patients (0-7 years old) during focus groups. Interview data were analyzed qualitatively and coded by an interdisciplinary group. An iterative approach identified overarching themes within the following Consolidated Framework for Implementation Research (CFIR) domains: outer setting, inner setting, and characteristics of individuals.

**Results:**

Overall, caregivers found the portal beneficial. Six themes emerged during thematic analysis. Outer setting themes were “lags and barriers to care” and “connections with outside providers.” Inner setting themes were “putting the ‘human’ in ‘human-centered design’” and “virtual access to information.” Characteristics of individuals themes were “flexibility” and “personalization.”

**Conclusions:**

The current study sheds light on caregiver perceptions of electronic portal use. Patient portals are an effective and well-received tool for patients with developmental delays and families accessing behavioral health information. Future targets for improvement include personalization, equitable access, and reach beyond single institutions.

## Introduction

Facilitating between-visit communication between pediatric care providers and patient caregivers improves patient outcomes across various pediatric conditions [1,2]. Effective and accessible mechanisms facilitate pediatric care provider and patient communication, particularly in the delivery of interdisciplinary care [3] given the multiple providers involved in coordinating care. Integrated primary care (IPC) links behavioral health care clinicians to medical physicians through the embedded delivery of prevention, assessment, and treatment services [4]. IPC services can be colocated appointments, delivered within the context of scheduled medical visits, and/or implemented through communications and supports between visits. Many integrated service delivery models rely on features within the electronic health record (EHR) to improve engagement, increase adherence, and open lines of communication between pediatric care providers and caregivers [5-7]. However, previous research has found that traditional primary care EHR systems may not have all the capabilities that behavioral health clinicians need, such as the ability to track behavioral health goals, administer electronic behavioral health screeners, and communicate effectively with other primary care physicians [8,9]. The absence of these functionalities may disrupt workflows and could lead behavioral health care clinicians to keep their own records outside of the EHR [10]. Modalities of virtual care have become widely used across health systems in the wake of COVID-19 and play a particularly important role in the delivery of behavioral health services [11].

With greater access to technology, patient care portals embedded within the EHR have been increasingly used as a mode of communication between pediatric care providers and caregivers. Embedded EHR portals are typically an “opt-in” electronic space where patients, patients’ caregivers, and pediatric care providers can communicate with other medical providers and staff regarding scheduling, nonurgent medical concerns, laboratory findings, orders, and referrals. It has been estimated that 60% of health care systems implement portals as an element of patient care and communication [12]. Patient adoption rates of portals were estimated to be 15% to 30% and growing following the COVID-19 pandemic, representing an area of promise for health care communication [12,13]. The American Academy of Pediatrics has highlighted the potential of EHRs to improve patient access to health information, facilitate preventative health, and support disease management [14]. In the context of IPC, portals can play an important role in screening; assessment; and facilitating aspects of care such as communication with multiple physicians involved in care, the provision of links to video visits, and a means of connecting patients with applications for symptom monitoring [8]. EHR portals tend to be well received by patients and caregivers, who endorse ease of use [15] and high ratings for satisfaction [16,17].

Despite the utility and ubiquity of patient portals in pediatric care, challenges exist in the uptake and reach of portal technology among underrepresented and marginalized patient populations [18]. Such inequities have been documented among economically disadvantaged and racialized groups [13,19]. For example, African American families and children on Medicaid are less likely to have a portal account set up when available [20]. There is limited research on the potential barriers to portal use among marginalized patient groups. Understanding how families navigate health care communication and their perceived benefit from technology in the delivery of health care may help make health care technology more accessible and user-friendly. Creating an EHR that works effectively for all medical and mental health care clinicians in integrated practices can be difficult [21]. Patient voices are often left out of the conversation when institutions make decisions about the adoption and implementation of patient-facing platforms [22], which may perpetuate or amplify existing disparities in pediatric behavioral health care [23]. User experience designs highlight that user voices can be leveraged to more readily align technologies with user needs, desires, and goals.

The current study aimed to begin filling in gaps in understanding marginalized patients’ and caregivers’ qualitative experiences with patient care portals when used in the context of IPC service delivery and developmental screening. We hypothesized that caregivers and families experience both barriers to and facilitators of portal-based care at the systemic, institutional, and individual levels. By identifying root causes of barriers and facilitators at each level, this work can inform the development of a multilevel toolkit to facilitate patient access to care and serve as a first step in research to refine EHR experiences for IPC.

## Methods

### Setting and Overall Study Design

This study was a collaborative effort that included clinician researchers across multiple service delivery divisions, including IPC, developmental behavioral pediatrics (DBP), and general pediatrics. This study took place in a culturally and ethnically diverse primary care clinic at a Midwest academic medical center. Most families served within the clinic depend on publicly funded insurance and face many barriers to care (ie, financial, social, logistical, and cultural). The setting included the use of a patient portal that allowed families to receive notifications about appointments, access clinical care through video visits, schedule and reschedule appointments, send and receive messages to and from pediatric care providers, complete behavioral health questionnaires before their visits, request their medical record for mental health appointments, submit release of information forms for outside medical and school providers to talk with third parties (eg, schools and mental health clinics), and access resources (eg, patient education materials and community mental health resources). A qualitative research design was used involving focus groups to explore participants’ perceptions and experiences, allowing for in-depth discussions and identification of shared themes and insights. We used the Consolidated Criteria for Reporting Qualitative Research (COREQ) guidelines to ensure a rigorous qualitative methodology [24]. Data were coded, analyzed, and interpreted using the Consolidated Framework for Implementation Research (CFIR) [25] to provide a structured approach for identifying key factors influencing the implementation of the patient portal across both internal and external systems of care.

### Procedures

#### CFIR

Focus group questions were developed, and qualitative analyses were conducted using the CFIR. The CFIR is a flexible framework that is adaptable to a variety of study methods. The CFIR includes 5 constructs: intervention characteristics, outer setting, inner setting, characteristics of individuals, and process. The study team identified 3 CFIR domains that were most relevant to this study: outer setting, inner setting, and characteristics of individuals. As this study did not analyze the effects of an intervention, the other 2 domains (ie, intervention characteristics and process) were not included in the qualitative analysis.

The focus group questions centered on facilitators and barriers in the outer setting, inner setting, and characteristics of individuals domains. Questions were open-ended and probed about caregivers’ experiences and preferences as well as facilitators of and barriers to their children receiving care, especially those related to the use of the patient portal. The focus group questions were developed using an iterative process: each interview informed the script for the following focus group to delve deeper into different areas mentioned by the previous groups.

#### Recruitment

We completed a retrospective chart review of the EHR from January 2023 to March 2023 to identify families with children aged 0 to 7 years who had received IPC or DBP care to inquire about participation in this study. Families from this chart review and other patients who were suggested by IPC and DBP providers were contacted via phone call to determine their interest in participating in this study. The IPC and DBP clinics were embedded in a racially and ethnically diverse clinic with approximately 66% of children served by Medicaid. This clinic is affiliated with a Midwestern academic medical center. Families were then approached via phone call with a research assistant and provided with study details and next steps for participation if interested. Our sample, therefore, included participants from multiple recruitment methods and a convenience sample of interested families. Caregivers who expressed interest in participating were given the option of taking part in in-person or virtual groups, and they provided their preference for time of day. Given that this study was institutional review board exempt, caregivers verbally assented to the study via phone and were enrolled after indicating that they would like to participate in the study.

Inclusion criteria were caregivers with 1 or more children receiving behavioral health services (IPC or DBP), aged 0 to 7 years, and who were perceived as having a behavioral or developmental concern needing more care as identified by a pediatric care physician. Exclusion criteria were caregivers who did not speak or understand English sufficiently.

In total, 28 caregivers were approached via phone about participating in this study, of whom 16 (57.1%) indicated that they would like to attend. Groups were formed by matching caregivers’ modality (ie, virtual or in person) and time of day (morning, midday, or afternoon) preferences. Three to 6 caregivers were scheduled for each focus group. Caregivers were contacted via phone to provide focus group details and confirm that the date and time worked for their family. A total of 87.5% (14/16) of the caregivers confirmed that they were able to attend one of the focus group offerings. The caregivers were called the day prior to the focus group to ensure that it still worked for their family. Of the 14 caregivers who confirmed, 7 (50%) completed interviews, and 7 (50%) who were scheduled for interviews did not attend. It is unclear why these 7 caregivers were unable to attend. The research assistant attempted to call the families 10 minutes after the focus group began to ask them whether they needed assistance logging on to the virtual link. The groups took place from January 2023 to April 2023.

#### Focus Groups

An in-person focus group and 2 virtual focus groups were led by a parent advocate and community partner trained in reflective practices, whereas the third virtual focus group was led by the research assistant. The parent advocate told the participants that their passion for helping on this project came from their own children with disabilities and their desire to help other families receive the medical care they needed for their own children. The in-person focus group took place at a local library, whereas the virtual focus groups were conducted from wherever the caregivers desired. The parent advocate and research assistant were given interview guides with sample questions and prompts that were created by the authorship team. Two children were also present at the in-person group along with 2 researchers. As the Zoom (Zoom Video Communications) focus groups took place wherever the caregivers desired, there may have been other individuals listening who were not directly involved in the focus group. Each participant engaged in a single focus group.

All focus groups were recorded and transcribed. The virtual focus groups were video recorded, whereas the in-person focus group only recorded audio. Field notes were taken both during and after the focus groups. Transcripts were not returned to participants for comment and/or correction. Interested caregivers were placed in focus groups based on availability and modality preference (in person vs virtual). A Zoom link was sent to the primary email of the caregivers participating in the virtual focus groups. After low attendance to the first virtual focus group, caregivers in the subsequent groups received a reminder email 1 week before, a reminder call within 48 hours of the focus group, and a call 10 minutes after the focus group began if they were not on the Zoom link to double-check whether the technology was working. In some cases, the Zoom link needed to be resent. After families were identified for inclusion in the study, a chart review was conducted to determine the children’s race and ethnicity, age, sex, and insurance provider.

### Ethical Considerations

This study was overseen by the University of Michigan Institutional Review Board (HUM00215154) and was deemed “not regulated.” Caregivers provided verbal consent upon scheduling the focus groups. At this time, they were informed that this was an optional qualitative improvement project and that they could leave or withdraw from the study at any time. Researchers deidentified transcripts; the transcripts were housed in a secure, protected health information drive; and any documents containing protected health information were password protected. Caregivers were also notified that their information would be deidentified when the findings were published. Participants received a US $60 Visa gift card for taking part in this study.

### Qualitative Analysis

The CFIR was used to categorize responses relevant to implementation in combination with grounded theory methods to allow novel themes to emerge. After each focus group, the research team met as a group and independently read the transcripts to discuss major themes that emerged from each relevant CFIR domain. A coding tree was developed within the CFIR domains, with 2 themes per domain. The research team consisted of 3 physicians (MD), 2 licensed psychologists (PhD), and 1 research assistant. Two of the physicians were developmental behavioral pediatricians, and 1 was a primary care pediatrician. The 2 PhD psychologists were integrated behavioral health psychologists. The research assistant was a PhD candidate in clinical psychology. All members of the research team identified as women, were from a diverse range of backgrounds (Asian, Black, Middle Eastern, and White), worked at an academic medical center, and had experience working on qualitative research within IPC. The participants knew that the researchers consisted of physicians and psychologists within an academic medical center. The focus group transcripts were read by all 6 researchers to create themes and identify example quotes. Two researchers (one of the physicians and one of the psychologists) returned to the transcripts to code for themes iteratively. Following each focus group discussion, we discussed the conceptual depth of themes and agreed through collaborative consensus. After completing 4 focus group interviews, it was decided that adequate conceptual depth and data saturation were achieved. Data were managed in Microsoft Excel. The outer setting domain involves how well the institution connects and communicates with outside referrals and agencies. For this study, many barriers were identified in the outer setting, including structural barriers to accessing the portal and portal resources. The inner setting domain details how well the institution connects and communicates within the organization. Many strengths were identified in the inner setting, including benefits and opportunities for portal use. The characteristics of individuals domain included aspects of caregivers’ own preferences or personality that aided or hindered portal use and their preferences for communicating with pediatric care providers through the portal. Many structural barriers existed for caregivers’ participation in this study, contributing to a small sample size. However, in the tradition of qualitative research, high information power was achieved through the narrow aim, dense specificity, use of an applied theory, strong dialogue, and case analysis [26].

## Results

### Overview

One in-person and 3 virtual 90-minute focus groups were hosted involving 7 caregivers of children 2 to 7 years of age. One focus group only had 1 caregiver attend, but the other 3 had multiple caregivers in attendance. All 7 caregivers were female. The children receiving behavioral health services were of Black (n=3), White (n=3), and other (n=1) races. (see Table 1 for demographic information). Participants provided feedback to the research team about clinic changes that they would like to see.

**Table 1:** Electronic medical record demographic information (N=7).

Variable		Participants, n (%)
Patient age (y)		
	2	1 (14.3)
	3	1 (14.3)
	4	1 (14.3)
	5	2 (28.6)
	6	0 (0)
	7	2 (28.6)
Child sex		
	Male	3 (42.9)
	Female	4 (57.1)
Child race		
	Black	3 (42.9)
	White	3 (42.9)
	Other	1 (14.3)
Insurance		
	Public	1 (14.3)
	Private	6 (85.7)

Caregivers generally described positive experiences with the portal, which helped them better access their children’s health care and connect with their behavioral health care clinicians. Caregiver access and comfort with their children’s patient portal was revealed as the most influential variable to their behavioral health care experience. Caregivers who could access their children’s EHR portal shared that they liked the ability to schedule and see appointments, receive appointment reminders, and communicate with pediatric care providers. Conversely, parents who identified portal access as a barrier reported difficulties using technology and limited access due to their children’s age and/or their caregiver status. Overall, 6 themes were identified, which we categorized into the outer setting, inner setting, and characteristics of individuals domains of the CFIR (Table 2).

**Table 2:** Themes and constructs with example quotes.

Construct		Description	Example quotes
Outer setting			
	Lags and barriers to care	This theme emerged as a concept that referred to systemic factors shaping caregivers’ access to care.	“...if you have to call for an appointment, a lot of times with the call center you have to wait or they transfer me to the wrong people and then I spend easily an hour on the phone trying to figure out what his appointment is or following up on a referral that didn’t get placed.” No portal access for foster parents: “And because they’re not fully adopted, they are not allowing me to use the portal. And not being able to use the portal is unfair from my standards....” Rescheduling leading to delays in services: “It could be long, long wait times to get another appointment back. It could be six weeks, it could be eight weeks.... And especially with early intervention, because with early intervention, every day lost is a day you can’t get back.”
	Connections with outside medical and behavioral health providers	This theme referred to caregivers’ desire for care coordination between medical and behavioral health providers using the EHR^a^ patient portal.	“I’m a fan. In fact, I think that there should be like a universal one [portal system], because it shouldn’t be.... That’s the one hassle I’ve had. And I get like why these privacy laws are there and whatnot.... Can we streamline this please and make life simple for everybody?”
Inner setting			
	Putting the “human” in “human-centered design”	This theme refers to the various ways in which the portal can be used as a tool for medical information, accessing pediatric care providers, and coordinating their children’s care.	“I do [use the portal], as much as possible. That’s why I don’t want to go see a doctor anywhere else because it makes my life easy.” Difficulties with setting up the portal: “What I don’t like about it is I just had my son and nobody has showed me how to sign up for the portal. I mean, they gave me a piece of paper, but I’m like, I’m a more show me type person, so show me how to access this patient portal.”
	Virtual access to information	This theme refers to the overarching ways in which families seek information on the internet to make medical decisions.	Parent describing behavioral health videos sent through the portal: “Because at least the video is easy to you just watch and you can follow what their actions. I mean, it’s just my own opinion.”
Characteristics of individuals			
	Flexibility	This theme emerged from caregivers’ desire to have a responsive medical system that suits each unique family system.	“I like in-person just ’cause I mean, I feel like if it’s relating to my kid, she probably needs to be present and she’s not going to be present for a virtual meeting. [Chuckle] That’s not going to happen.” “And she called me at home before the holiday and said, ‘I just wanna make sure, is there anything else? Did you think about what I said? Is there anything more for you? How he is....’ And my heart just melted. I said she didn’t have her nurse or you know, some other person. She called me. I felt like so great.”
	Personalization	This theme emerged from caregivers’ need for tailored and individualized care.	“That’s an interesting question. I think it’s probably situational specific because what if it’s something I have questions about, or what if it’s something that requires a dialogue for understanding? Because this is all.... Even though I’m in it for like two and a half plus years now, it’s still new territory to me. I’m learning every day about my child and what she needs and how to help her and how to deliver it, and the best delivery method and mode that she responds to that’s best for her. So if it’s like a social activity I would almost think social instruction would be, whether it’s over the phone or by video...would be super beneficial. And but if it was something more like, little tips or tricks for improving fine motor skills, I would see how a worksheet delivered through the portal would be suffice.”

^a^EHR: electronic health record.

### Outer Setting Themes

In the outer setting, themes identified were “lags and barriers to care” and “connections with outside medical and behavioral health providers.” Lags and barriers to care emerged as a concept that referred to systemic factors shaping caregiver access to care. Caregivers generally viewed the portal favorably to reduce the lags and barriers to care, such as allowing for easy scheduling and rescheduling of appointments, but some families were not provided with equitable access, especially those providing care for foster children. Some of the lags and barriers to care were mentioned 11 times during the focus groups and included long waits for the call center, no portal access for foster parents, and delays in care when parents needed to reschedule appointments. The lags and barriers to care were perceived as critical. For example, a caregiver stated the following:

...with early intervention, every day lost is a day that you can’t get back.

As represented by the theme “connections with outside mental and behavioral health providers,” caregivers reported a strong desire for care coordination between pediatric care providers using the portal and included difficulties and lengthy intake processes with outside referrals as major barriers to care. Outside medical and behavioral health providers included Early On, applied behavior analysis therapy, behavioral therapy, community mental health, and other outside behavioral or developmental services. One caregiver wished there was a “universal” portal system:

Can we streamline this process and make life simple for everybody?

Connections with outside medical and behavioral health providers were mentioned 6 times during the focus groups.

### Inner Setting Themes

In the inner setting, the themes identified were “putting the ‘human’ in ‘human-centered design’” and “virtual access to information.” The “putting the ‘human’ in ‘human-centered design’” theme refers to the various ways in which caregivers can use the portal as a tool for medical information, accessing pediatric care providers, and coordinating their children’s care. This theme included statements about how the portal is an effective tool and ways in which it could be more tailored to meet families’ needs. One caregiver even noted the following:

I do [use the portal], as much as possible. That’s why I don’t want to go see a doctor anywhere else because it makes my life easy.

The theme of putting the “human” in “human-centered design” was identified 8 times throughout the focus groups. When 1 caregiver had difficulty getting ahold of someone to help them log into the portal, they mentioned missing behavioral health care visits. Virtual access to information refers to the overarching ways in which families seek information online to inform medical decisions. It was identified 12 times during the groups and included how caregivers access information about their children’s health care, including information sent through the portal, such as websites with behavioral health information, and a desire for behavioral health providers to send behavioral health parent training videos. Without information provided through visits or the portal, parents stated that they would refer to social media.

### Characteristics of Individuals Themes

Themes identified in the characteristics of individuals domain were “flexibility” and “personalization.” Flexibility was conceptualized as the desire to have a responsive medical system that suits each unique family system and was mentioned 6 times during the focus groups. Some parents preferred visits to be in person, whereas others preferred them to be virtual. One caregiver mentioned that it was very challenging to bring her child to an in-person behavioral health visit, whereas another caregiver mentioned that it was difficult to get her child to sit still for a virtual behavioral health visit. Personalization was conceptualized as the caregiver’s need for tailored and individualized care for their child and was mentioned 10 times during the focus groups. One caregiver noted the following:

Well, I know what’s best ’cause I’m the parent. I’m the one dealing with it. But I want them [medical and behavioral health providers] to feel heard and I wanna feel heard. I wanna come to a good compromise.

Whatever method of communication (in person, virtual, phone, or portal message), parents want personalized care that fits their children and families. Caregivers seek flexibility and personalization in their care, and the portal can either help facilitate these goals or hinder them depending on how it is designed and used.

## Discussion

### Principal Findings

To our knowledge, this is the first study of its kind to use the CFIR to better understand caregiver-reported facilitators and barriers surrounding accessing and using patient care portals in the context of pediatric IPC. The purpose of this investigation was to gather information for the development of a streamlined behavioral health toolkit that will guide institutional policies and procedures surrounding the use of patient-facing EHR tools to improve patient care. The current study is the first to our knowledge to examine caregiver perspectives on the use of a patient EHR portal for pediatric IPC, finding that caregivers believe it is a useful tool but there continue to be access issues to the portal.

Overall, the caregivers included in this study viewed the patient care portal as a positive space to participate in care for their children, in particular, helping meet needs within the context of the health system (inner setting). Structural barriers and inequities in caregiver access to electronic behavioral health records were also reported across participants, particularly surrounding care coordination and the need for more tailored and individualized care.

Using the family voice in our study design allowed for the identification of human-centered design principles to inform potential improvements in the patient and caregiver experience. Caregiver-reported positive interactions with the portal fell primarily within the inner setting domain of the CFIR, which suggests that, at the individual patient level, the portal aids in effective communication between caregivers and pediatric care providers. Specifically, the patient care portal was helpful in aiding caregivers’ scheduling of appointments and their access to clinical care services such as video visits. Families also reported accessing health information on social media and through other open online sources, suggesting that future portal integration of health care information from trusted resources may be an area that families may find beneficial. Ultimately, families perceived the patient portal as an effective bridge to health care access. However, families often differed in their preference for virtual vs in-person care and communication of behavioral health needs.

A structural barrier commonly reported by participants was the problem of lengthy wait times to be able to schedule or reschedule behavioral health services when having to go through call centers. The patient portal, however, was perceived as something that helped increase access to care and reduce wait times. Prior work using time use diaries has found that low-income families are more likely to spend an additional 12 minutes per day waiting for services compared with their higher-income counterparts [26]. One potential mechanism might include a dearth of medical and behavioral health providers accepting Medicaid [27], which limits caregiver access to medical care overall. In this study, families indicated that the patient portal was one way to bypass long waits in the call center and that it was the more ideal way to reschedule care. Patient portals may be an important way to accommodate the time constraints of low-income families, whose employment may not have substantial flexibility to address scheduling and make phone calls during regular clinic hours.

Another area of potential future innovation that should be considered is developing capabilities to allow for communication across pediatric care providers and institutions. Families noted that systems-based communication between pediatric care providers and early intervention or community mental health was lacking, but a portal system that was universal and not institution specific could facilitate warm handoffs and referral monitoring between disparate systems. For example, the patient portal could display referral status across systems to create continuity and personalization of care, which families valued and desired.

Finally, an important barrier identified by caregivers was the lack of universal accessibility of the patient portal to foster parents. The number of children in foster care has increased from approximately 397,000 in 2012 to 442,000 in 2017 [28]. Children in foster care experience increased health problems compared to the general population and may require more extensive communication with their pediatric care providers [29]. For this population, there may be multiple encounters with the health care system in many settings and with many caregivers, leading to fragmented care [30] and complicating the communication of critical health information [31]. There is promise in leveraging the patient portal to create a more streamlined system to detangle health care delivery among children in foster care. Recognizing this gap in service delivery, Texas; Cincinnati, Ohio; and Allegheny County, Pennsylvania, have passed laws or created data systems that improve health care communication for children in foster care. Lessons learned from these regional efforts could be used to inform how health systems can incorporate more inclusive policies to allow foster parents to have portal access for services and features that can facilitate patient care while maintaining protection and privacy of patients in state custody.

Our study had several limitations. As a single-center study, our work may not be generalizable to other populations using different EHR portal systems or in other states with different regulations regarding foster care or privacy. In addition, data were qualitative and included a small number of participants. Patient perspectives may not reflect the views of other populations. Strengths of this study include the specificity of behavioral health information and inclusion of a low-income and racialized population, who experience greater barriers to health care and access. Future research should explore caregiver education on features and functions of the patient portal, as well as how policy changes can help caregivers better access behavioral health information, provide a more positive experience regarding scheduling appointments, and be more inclusive.

### Conclusions

Using in-depth qualitative methods, we found that a patient portal is an effective and well-received tool for patients accessing behavioral health information. Despite positive perceptions, ways in which these tools can be personalized and reach beyond single institutions were identified as potential areas of improvement. It was also evident that disparities remain in health IT access that require systemic work to ensure that all populations have access to high-quality care, streamlined communication, and behavioral health information.

## Data Availability

Data are available from the corresponding author ETK on request (lizkoval@iu.edu).

## Abbreviations

CFIR: Consolidated Framework for Implementation Research
COREQ: Consolidated Criteria for Reporting Qualitative Research
DBP: developmental behavioral pediatrics
EHR: electronic health record
IPC: integrated primary care
